# Case report of Tako-Tsubo cardiomyopathy associated with repetitive anaesthesia in a female patient with Tako-Tsubo cardiomyopathy

**DOI:** 10.1186/s12871-015-0022-z

**Published:** 2015-03-27

**Authors:** Jochen Hinkelbein, Christian Mey, Gerrit Brinker, Roman Pfister, Bernd W Böttiger

**Affiliations:** 1 Department for Anaesthesiology and Intensive Care Medicine, University Hospital Cologne, Kerpener Str. 62, 50937 Cologne, Germany; 2 Department for Neurosurgery, University Hospital Cologne, Kerpener Str. 62, 50937 Cologne, Germany; 3 Department of Cardiology, University Hospital Cologne, Kerpener Str. 62, 50937 Cologne, Germany

**Keywords:** Tako-Tsubo cardiomyopathy, Broken heart syndrome, Transient left-ventricular apical ballooning syndrome, Stress-induced cardiomyopathy, Ampulla cardiomyopathy

## Abstract

**Background:**

Tako-Tsubo cardiomyopathy (TTC) is a rare disorder with high relevance for anaesthesia. It is an acute cardiac syndrome characterized by an acute onset of reversible left ventricular dysfunction associated with emotional and physical stress. This is the only case published of a patient having five severe Tako-Tsubo incidents in five consecutive general anaesthesia procedures within one year.

**Case presentation:**

A 61 years old female patient (height 1.65 m; weight 70 kg) presented with a haemorrhagic pituitary adenoma with compression of the optic chiasm and was scheduled for transnasal endoscopic tumour resection. We report a case series with five consecutive anaesthesia procedures in the same patient for neurosurgery.

This case series is remarkable since the severe symptoms occurred during every anaesthesia procedure. The female patient was resuscitated two times including therapeutic hypothermia, but fortunately no neurological or cognitive deficit was detectable.

**Conclusions:**

TTC may initially present in the perioperative period with pulmonary oedema, electrocardiographic (ECG) changes, elevation of cardiac enzymes, and cardiogenic shock or cardiac arrest.

Since the risk of recurrence is considered to be low in TTC, this case report is of high interest. In each procedure similar clinical signs were found which resulted in severe haemodynamic derangements in every manifestation and cardiac arrest in two of the manifestations. Despite cardiopulmonary resuscitation twice, the patient survived without any neurological deficiency.

## Background

Tako-Tsubo cardiomyopathy (TTC) is a rare disorder with an annual incidence of approximately 0.00006% [1]. This syndrome was first described in Japan in 1990 [2].

It is also known as “takotsubo syndrome” [3,4], “broken heart syndrome” [4,5], “transient left-ventricular apical ballooning syndrome” [5-7], “stress-induced cardiomyopathy” [5,8], “ampulla cardiomyopathy” [4,8], or “stress-induced myocardial stunning” [9,10]. Although the syndrome is usually induced by a sudden stress reaction, symptoms may also occur without any detectable cause.

**Figure 1:**
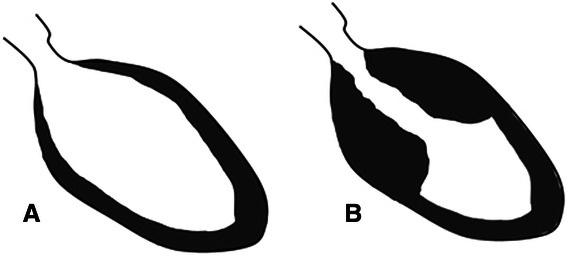
**Normal heart (A) and Tako-Tsubo sign (B) with apical ballooning (end-systolic view).**

The syndrome usually manifests like acute myocardial infarction but is characterized by generally reversible left ventricular dysfunction with typical akinesia of the apical segments and hypercontractility of the basal segments and is associated with emotional or physical stress (Figure 1) [5,7,10].

It occurs most often in post-menopausal women and it is usually triggered by emotional or physical stress, with complete recovery of left ventricular systolic function within a few days or weeks. For diagnosis, significant coronary artery disease has to be absent [5]. The diagnosis of TTC should be based on the following Mayo Clinic criteria [11]: (a) transient akinesia or dyskinesia of left ventricular apical and/or midventricular segments; (b) no angiographic evidence > 50% coronary artery stenosis, or plaque rupture, or intracoronary thrombus formation; (c) new electrocardiographic (ECG) abnormalities (dynamic ST-T changes or T-wave inversion); (d) absence of intracranial bleeding, phaeochromocytoma, and myocarditis.

Tako-Tsubo cardiomyopathy may initially present in the perioperative period with pulmonary oedema, ECG changes, elevation of cardiac enzymes, and cardiogenic shock or cardiac arrest [7,12]. Many reports show the onset in the preoperative [13] and postoperative period [3,7,12,14,15] without an association to anaesthesia. Only few onsets are reported to happen in the intraoperative period [7,16,17], during anaesthesia or anaesthesia induction.

The risk of recurrence is generally considered to be very low [6]. Yet there are two case series published reporting of three general/regional anaesthesia procedures in patients with Tako-Tsubo cardiomyopathy [7,18].

Although extensive literature research was performed by our team, we did not find any other case series of patients receiving repetitive anaesthesia. Here we report on a case of a female patient, having 5 severe TTC incidents in 5 consecutive procedures under general anaesthesia within sixteen months.

### Consent

Since this report is a case report, no ethical committee statement is available. Written informed consent was obtained from the patient for publication of this Case report and any accompanying images. A copy of the written consent is available for review by the Editor of this journal.

## Case presentation

**Figure 2:**
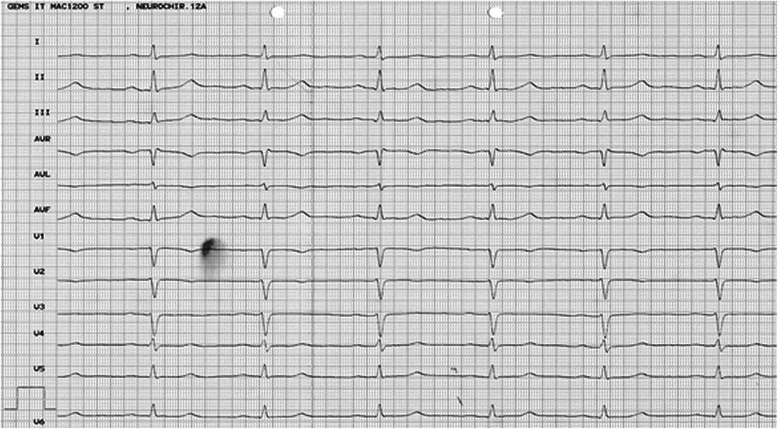
**Resting ECG of the patient.**

A 61 years old female patient (height 1.65 m; weight 70 kg) presented with a haemorrhagic pituitary adenoma with compression of the optic chiasm and was scheduled for transnasal endoscopic tumour resection. During the preoperative consultation by an anaesthesiologist, she reported of intermittent extrasystolia why she underwent coronary diagnostics two years before. At that time diagnostics were uneventful and coronary heart disease (CHD) had been excluded by coronary angiography. Preoperative anaesthesia evaluation including ECG (Figure 2) and blood analysis was unremarkable. Both physical examination and history were uneventful (last surgery >20 yrs before without known problems).

### Anaesthesia #1 (March 12, 2012)

Surgery was scheduled and the patient was considered for general anaesthesia. The patient was pre-medicated with midazolam 7.5 mg orally prior to arrival in the OR. Basic monitoring (ECG, pulse oximetry and non-invasive blood pressure (NIBP) measurement) was applied and a peripheral venous line was installed. For induction of anaesthesia, sufentanil 70 μg, propofol 150 mg, and atracurium 50 mg i.v. were used. During induction the endotracheal tube was placed successfully in the second attempt by the anaesthetist due to a slightly difficult airway (Cormack & Lehane Score, C&L III). The initial course of anaesthesia was uneventful except for a bradycardia (27 bpm) which was treated with 0.5 mg atropine intravenously (i.v.). During the surgical transnasal approach, the patient suddenly developed significant ST segment elevation followed by ventricular fibrillation (VF) being treated by basic and advanced life support (BLS, ALS) for 5 minutes including the intermittent use of intravenous epinephrine and one single defibrillation. Thus, surgery was aborted before starting tumour resection and the patient transported for coronary angiography. Since no coronary heart disease (CHD) was found and the ventriculogram showed typical regional wall motion abnormality with apical ballooning, the diagnosis of Tako-Tsubo cardiomyopathy was stated according to the Mayo Clinic guidelines [11] by a specialist for cardiology. The further course on the Intensive Care Unit (ICU) was uneventful and the patient was extubated several hours later. There was no neurological or cognitive deficit detectable. At day 2 the patient was discharged from ICU to the normal ward without wall motion abnormalities (trans-thoracic echocardiography, TTE). Since surgery had to be aborted without initiating resection of the tumour, it was scheduled two months later. The patient was asymptomatic in the meanwhile.

### Anaesthesia #2 (May 4, 2012)

To prevent potential recurrence of TTC, the complete team was instructed prior to anaesthesia and surgery. The patient was pre-medicated with midazolam 7.5 mg orally prior to arrival in the OR. Basic monitoring was applied and a peripheral venous line was installed. For induction of anaesthesia, sufentanil 50 μg, propofol 200 mg, and atracurium 30 mg i.v. were used. Both induction of anaesthesia and the course of surgery were uneventful except for an intermittent ST-segment elevation and an accompanying hypotension which were controlled by a single bolus injection of norepinephrine (20 μg). The further course of surgery and hospital stay presented without any abnormalities.

### Anaesthesia #3 (Aug 28, 2012)

Again, transnasal surgical decompression became necessary because of development of a symptomatic intra- and suprasellar cystic lesion highly suspicious of an abscess at the former tumour site. The patient was pre-medicated with midazolam 7.5 mg orally prior to arrival in the OR. After applying basic monitoring and a peripheral venous line, an arterial cannula was inserted into the patient being awake. For induction of anaesthesia, sufentanil 50 μg, propofol 180 mg, and atracurium 40 mg i.v. were used. Right after intravenous induction of anaesthesia and well before surgical preparation and positioning of the patient, again, the patient suddenly developed significant ST segment elevation followed by a pulseless electrical activity (PEA) and intermittent VF which were, again, treated by basic and advanced life support (BLS, ALS), epinephrine, and defibrillation. Return of spontaneous circulation (ROSC) was achieved after 30 minutes of cardiopulmonary resuscitation (CPR). Due to suspicion of an acute myocardial infarction, anti-coagulative treatment was started with acetylsalicylic acid along with heparin.

Since haemodynamics were insufficient and extensive inotropic and vasopressor support was required (norepinephrine 1,000 μg/h and epinephrine 2,500 μg/h by a syringe pump), cannulation for emergency extracorporeal membrane oxygenation (ECMO) was discussed but at last not performed due to increasing haemodynamic stability to a tolerable level. Since inotropic support was steady but still high-level at this time, surgery was cancelled and the patient transported to ICU for induction of therapeutic hypothermia and improvement of the haemodynamic situation, which was successful.

The further course on ICU was uneventful and, therefore, surgery was performed 8 days later after full recovery of the cardiac function (trans-thoracic echocardiography) without any problems in the still sedated and ventilated patient. A sellar abscess could be confirmed and was treated by surgical evacuation and postoperative antibiotics. After surgery, the patient was extubated and discharged from ICU at day 12. Again, there was no neurological or cognitive deficit detectable.

### Anaesthesia #4 (Feb 25, 2013)

In spite of adequate surgical and medical therapy of the primary lesion, a recurrence of the abscess was detected several months later. The patient was necessarily presented again for anaesthesia and surgery. In order to provide a maximum of safety for the patient, extensive, interdisciplinary preoperative arrangements were made between anaesthetists, nurses, and surgeons (transfer to the operating room (OR), two experienced senior specialists for anaesthesia, medical equipment, monitoring devices, postoperative, and crisis management).

The patient was orally pre-medicated with lorazepam 1 mg at the evening before surgery. On the day of surgery, she received an additional oral pre-medication of 7.5 mg midazolam and was transferred smoothly to the OR. Basic monitoring was applied and a peripheral venous line was installed. For induction of anaesthesia, midazolam 5 mg, sufentanil 50 μg, propofol 50 mg, and atracurium 50 mg i.v. were used. Endotracheal intubation by direct laryngoscopy was difficult due to reduced visibility on the vocal cords (Cormack & Lehane III), but successful in the second attempt performing backwards upwards rightwards pressure (BURP) and reclination of the head. In parallel the second anaesthetist inserted an arterial line and placed the transoesophageal echocardiography probe (TEE) for haemodynamic and cardiac monitoring. In addition, a central venous catheter was inserted into the left subclavian vein.

**Figure 3:**
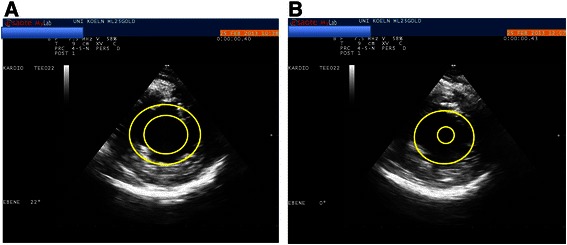
**Original case TEE sequence in anaesthesia #4. A**. acute onset of left ventricular dysfunction/considerable hypokinesia (reduced contractility: outer circle: diastole; inner circle: systole). **B**. after successful therapeutic intervention: normal left ventricular function/no considerable wall motion abnormalities (normal contractility: outer circle: diastole; inner circle: systole).

After induction of anaesthesia within preparation for surgery, the patient developed hypotension followed by ST segment elevation. Simultaneously the apparently etiologic sudden onset of left-ventricular hypokinesia could be detected by TEE (Figure 3A). This considerable haemodynamic depression was instantly treated by application of a fluid bolus and repetitive vasopressor support (Σ 100 μg norepinephrine i.v.). Within few minutes haemodynamics stabilized and the described echocardiographic symptoms of left-ventricular function disorder diminished (Figure 3B). Surgery, again by transnasal endoscopic abscess evacuation, and the further course of anaesthesia were uneventful except for another slight period of discrete echocardiographic signs of the syndrome intraoperatively. Since symptoms were recognized early and treated sufficiently, they diminished 1 min. after appearance. In particular, mean arterial blood pressure was treated to remain above 70 mmHg using continuous low-dose application of norepinephrine with a syringe pump. Although prepared for instant application, neither inotropic support (in terms of milrinone and/or epinephrine continuously) nor CPR was required in anaesthesia #4 even in the acute appearance of the cardiomyopathy syndrome since haemodynamics never dropped to intolerable levels. After surgery the patient was transferred to the ICU and was extubated 3 hours later. The further course of medical treatment was completely uneventful and the patient was discharged to the normal ward at day 3.

### Anaesthesia #5 (Jul 2, 2013)

Due to recurrent rhinoliquorrhea out of the sellar floor at the former surgery site and failure of conservative treatment, another mucosal reconstructive surgery was required. In this case, preparation and preoperative management were identical. Anaesthesia was performed by the same team as in anaesthesia #4 accompanied by equal logistic and infrastructural preparations.

For anaesthesia induction again midazolam 5 mg, sufentanil 50 μg, propofol 50 mg, and atracurium 50 mg were injected. Endotracheal intubation by direct laryngoscopy was known to be difficult (Cormack & Lehane III) but easily possible with the available video laryngoscope. In parallel an arterial line for haemodynamic monitoring was placed by the second anaesthetist without problems in the right femoral artery (not palpable in the radial arteries) and a central venous catheter was installed. The transoesophageal echocardiography probe for cardiac monitoring was simultaneously positioned.

Shortly after induction of anaesthesia the patient developed hypotension, followed by bradycardia, ST-segment elevation, and contractile dysfunction (left ventricular hypokinesia as seen in the TEE similar to Figure 3A). These symptoms resembled the situation described in anaesthesia #4 but haemodynamic depression and contractile dysfunction were more pronounced and required intermittent vasopressor and inotropic support as well as a fluid bolus (∑ approx. 1 mg norepinephrine and 100 μg epinephrine). Systolic blood pressure never dropped below 50 mmHg and symptoms diminished within few minutes without the necessity for CPR since they were recognized early (TEE and arterial line) and treated sufficiently. Further treatment equalled anaesthesia #4 (continuous norepinephrine for MAP strictly < 70 mmHg and prepared milrinone) and the surgical period and the course of anaesthesia were completely uneventful. After surgery, the patient was transferred to the ICU and was extubated 2 hours later. The further course remained uneventful and the patient was discharged to a normal ward at day 3.

### Case discussion

The annual incidence of Tako-Tsubo cardiomyopathy is extremely low (approximately 0.00006% [1]) and the recurrence of a Tako-Tsubo syndrome is infrequent. This case series describes five repetitive anaesthesia procedures in the same patient within sixteen months. Our reported series shows are current Tako-Tsubo syndrome with similar clinical signs which were severe in every single case.

Anxiety about surgery or anaesthesia can be an inciting factor [13]. Benzodiazepines may be a good choice for sedation. However, more important as the drug itself, it is required to obtain adequate pre-medication prior to anaesthesia. A.K. Wong and colleagues presented a case given only 1 mg of midazolam preoperatively which resulted in an acute onset of Tako-Tsubo syndrome in their patient [13]. Therefore, sufficient sedation and anxiolysis in patients prior to scheduling for anaesthesia seems to be beneficial. In our case, we have chosen to use lorazepam and midazolam preoperatively in high doses to prevent anxiety and stress.

Besides preoperative anxiolysis, deep anaesthesia during intubation seems also to be beneficial. M. Brucoli and colleagues hypothesized that stress caused by superficial anaesthesia, insufficient analgesia, and surgical pain stimulus created a stressful event that can cause a potentially life-threatening catecholamine release [1]. Also the mechanism of TTC during anaesthesia or surgery could be a neuro-cardiac interaction. For this reason, several mechanisms can be entangled, part of the complex brain-heart connection. Although our patient was in deep anaesthesia (at least in cases #4 and #5), she developed the Tako-Tsubo symptoms after endotracheal intubation but before surgery has started. Additionally, our case was complicated by a moderate difficult airway (C&L III) which was handled by video laryngoscopy (#5) to prevent multiple intubation attempts.

Some authors postulated that, if a Tako-Tsubo cardiomyopathy is present, patients should be monitored by invasive arterial measurements, placed in the awake patient. However, this is questionable since insertion of an arterial cannula may also result in severe symptoms due to stress and pain [7]. In the cases #2 and #3 an awake arterial cannula was placed, but not in cases #4 and #5. We had an extensive team discussion on this topic prior to #4 and while working with two experienced specialists for anaesthesiology, we chose placing the arterial line after induction of anaesthesia to prevent unnecessary stress. However, typical symptoms occurred after induction of anaesthesia.

The major pathophysiological phenomenon involved in TTC is considered as disproportionate catecholamine discharge in response to stress. This catecholamine excess is thought to stun the myocardium. In contrast, milrinone is known as a non-catecholamine inotropic drug improving myocardial contraction by inhibiting type III phosphodiesterase. The initial choice of haemodynamic support with norepinephrine is, therefore, perhaps debatable as the Tako-Tsubo syndrome is related to exaggerated sympathetic activation, and increased catecholamine intake such as norepinephrine infusion could worsen symptoms [17]. Milrinone, on the other hand, decreases the systemic vascular resistance and pulmonary capillary wedge pressure. Furthermore, there is a lower increase in the heart rate and myocardial oxygen consumption with milrinone compared with catecholamines. But, the induced peripheral vasodilatation by milrinone may promote hypotension and may aggravate shock. Nevertheless, we have chosen a combination of norepinephrine and epinephrine for inotropic and vasopressor support which was clinically sufficient to treat symptoms.

In two of the presented five cases, CPR was required (5 min vs. 30 min), resulting in a very unstable haemodynamic situation. Therefore, it might be beneficial to have an intra-aortic balloon pump (IABP) available for haemodynamic support. In our case #3, haemodynamic situation after 30 min of CPR was very unstable. Installation of emergency extracorporeal membrane oxygenation (ECMO) was discussed interdisciplinary but surrendered.

It is remarkable that severe TTC symptoms in this female patient were found during every anaesthesia procedure. Additionally, although she was resuscitated two times (in case #3 with therapeutic hypothermia for 2 days), there was no neurological or cognitive deficit detectable.

Recommendations for anaesthesia in patients with severe TTC:

- Anaesthesia and surgery should be performed in large centres (sufficient personnel).
- Proper preoperative diagnostics is required. Check, if coronary catheterization is necessary.
- ICU must be available.
- Discuss crisis management and make interdisciplinary agreements.
- The anaesthesiologist should be experienced, should know TTC in detail and know how to react in case of a problem.
- Anaesthesia should be performed by two experienced anaesthetists
- Adequate medical premedication at the evening before surgery and the morning of surgery.
- Patient should be transported to OR without stress, preferably first position (under deep sedation and anxiolysis)
- Consider arterial line in the awake patient, but when anaesthetized seems more beneficial
- Perform deep anaesthesia for endotracheal intubation
- TEE should be used for detection of Tako-Tsubo symptoms since a dynamic left ventricular outflow tract obstruction (LVOTO) is reported in TTC-associated cardiogenic shock. Maintain good left ventricular preload to avoid LVOTO.
- Possibility to place urgently an intra-aortic balloon pump (IABP)
- ß-blockers seem to have beneficial effects

### Literature review

#### Recent literature published

The term “Tako-Tsubo” was initially used, because the morphological feature of a left end-systolic ventriculogram shows a balloon-shaped, short-necked, round flask resembling a Tako-Tsubo, a device used to trap octopuses in Japan (Figure 1) [19].

A literature search in Medline™/PubMed™ revealed only 19 hits for “TakoTsubo” and “anaesthesia” and 66 hits for “Takotsubo” and “anaesthesia” starting in the year 2002. When using “broken heart syndrome” and “anaesthesia” or “anaesthesiology” a total of 63 vs. 31 hits were found. There were no randomized controlled trials (RCT) found on this topic.

### Prevalence, pathogenesis, and prognosis

Today, the exact prevalence of the Tako-Tsubo syndrome is not known [20]. It is estimated at 2–8 per year and per 1 million inhabitants. Altogether more than 1,000 cases have been reported [21] but only few during anaesthesia. The syndrome was reported both in male [9,22] and female patients, but most reported cases of this syndrome (more than 90-95%) affect elderly women [7] being older than 60 years [22] or being postmenopausal [1,5,8,20]. The average age at diagnosis is 68 years [21]; however, a few cases have been described in paediatric patients [21].

Although the pathogenesis of Tako-Tsubo cardiomyopathy in not completely understood, catecholamine-mediated myocardial stunning due to enhanced sympathetic activity is the most widely accepted underlying mechanism [23]. From a clinical point of view, this syndrome can be similar to the acute coronary syndrome in awake patients which is the major differential diagnosis. Because the precise aetiology of the Tako-Tsubo cardiomyopathy remains unclear, there is no consensus for treatment during the acute phase [7].

Transient life-threatening complications were occasionally reported, including pulmonary oedema, cardiogenic shock, dynamic intraventricular obstruction, ventricular arrhythmias or ventricular fibrillation, rupture of the left ventricular wall, and death [19].

Although most patients recover without complications after an acute episode of Tako-Tsubo cardiomyopathy, 15%-45% of patients with an acute onset of symptoms develop cardiogenic shock and require inotropic and vasopressor support [4,6,8]. Other severe complications as ventricular fibrillation or cardiac arrest as well as death result in up to 21% of cases, respectively [4,13]. However, the patient’s initial prognosis is generally good after appropriate treatment of acute phase complications [19]. Nevertheless, in-hospital mortality is estimated with approximately 1.1% to 2.0% (range 0%-8% [13]) [6,21].

Following an acute episode, left-ventricular dysfunction usually resolves within several days or weeks [7] and an almost complete recovery is seen in 96% of the patients [24]. The prognosis of patients with TTC is usually good [7,10,12]. The risk of recurrence of this syndrome is low, and estimated at 0% to 11.4% up to 4 to 5 years after the initial episode [6,12,13,24], but it is unpredictable.

How to best prevent perioperative Tako-Tsubo cardiomyopathy and whether there is a best anaesthetic technique to use in patients with necessity for surgery who have experienced the syndrome previously is still unclear [4].

### Anaesthesia considerations

In several series, about 13% (6% to 16%) of cases occurred perioperatively [4,24]. Anxiety about surgery or anaesthesia can be an inciting factor [13]. No established guidelines exist neither for diagnostic strategy and risk stratification nor for treatment of patients with Tako-Tsubo cardiomyopathy [25]. Therefore, the ideal anaesthetic management of patients with TTC is unknown [7]. In combination with the rarity of the syndrome and thus the likeliness to become confronted with a first onset of the syndrome unprepared clearly makes it a challenge even for experienced anaesthesiologists [12]. This is more so, since the pathogenic correlates are less easily identified during anaesthesia [16].

Perioperative management strategy consists of both prophylactic and therapeutic components. One principle goal is to avoid psychological and physical stress that could trigger an acute episode of the cardiomyopathy [7,10,12]. The resulting centre column of prophylaxis is a sufficient anxiolysis which requires both psychological and pharmacologic approaches [7].

Additionally, an extensive anaesthesia anamnesis and information of the patient is essential. Besides ß-blocker therapy, there is no evidence to support any specific management strategy or drug therapy for the prevention of onset or recurrence. However, providing a rather deeper level of anxiolysis and sedation prior to transportation to the operating room might be beneficial [13].

Although it was suggested that regional anaesthesia (RA) may be a good alternative to general anaesthesia (GA) [7], this question is not finally answered and there is no consensus yet [7].

There were several drugs used for anaesthesia both in our case and other case reports (Table 1) and a definite connection between specific drugs and the emergence of acute TTC symptoms is not known. Laryngoscopy and intubation may cause the specific symptoms [17] and should therefore be brief to minimize sympathetic stimulation. ECG changes including dynamic ST-elevation should be carefully monitored [7].

**Table 1:** **Previously used anaesthesia drugs in patients with Tako-Tsubo cardiomyopathy**

Drug group	Substance	Citation
Sedation	Midazolam	[7]
Analgetics	Fentanyl	[3,7,16]
	N_2_O	[7]
	Sufentanil	[17]
Hypnotics	Propofol	[3,7,16,17]
	Sevoflurane	[3,7,16]
Muscle relaxants	Rocuronium	[3,7]
	Cisatracurium	[16]

Smooth emergence and extubation are also essential to avoid increases in catecholamines that may trigger these cardiac symptoms [7,14]. Postoperatively, patients should stay in the intensive care unit (ICU) for vigilant monitoring and adequate pain control because symptoms frequently occur in the postoperative period [12,14].

### Acute episode

Although atropine was administered to treat resulting symptoms in some case reports [9] and in one of our cases, it is usually not recommended during symptomatic Tako-Tsubo cardiomyopathy [23]. The withdrawal of parasympathetic drive in such cases should exacerbate sympathetic activity, leading to the genesis or worsening of disease activity [23]. This effect was observed in some cases [9,23].

Maintenance of a sufficient mean arterial pressure to secure adequate coronary perfusion is essential and may demand the application of vasopressors. Inotropic support may be necessary but has to be monitored carefully since stimulation of β_1_-receptors by endogenous catecholamines is one of the discussed pathogenetic factors in the development of TTC. Also a dynamic obstruction in the left ventricular outflow tract (LVOT) similar to systolic anterior motion (SAM) may occur or be aggravated by inotropic agents when administered without echocardiographic monitoring.

A dynamic left ventricular outflow tract obstruction (LVOTO) is reported in TTC-associated cardiogenic shock [26]. Indeed, low left ventricular diastolic filling pressure, hyperdynamic motion of basilar walls, eventually pre-existing basal septal hypertrophy, and inappropriate catecholamine treatment could all cause dynamic LVOTO. For this reason, maintaining good left ventricular preload and reducing dynamic LVOTO could be the main therapeutic target in TTC cardiogenic shock. Complete haemodynamic monitoring integrated with TEE is essential to establish proper management and avoid unnecessary or harmful pharmacological intervention.

Therefore, both transthoracic and transoesophageal echocardiography mark an important monitoring component as they can identify regional wall motion abnormalities of the left ventricle [3] and therefore guide specific therapeutic interventions and directly show their effects. They should thus be a standard during anaesthesia in these patients.

## Conclusion

Tako-Tsubo syndrome is an adverse event that may occur at induction of general anaesthesia, whose relationships with anaesthesia and surgical stress should be studied because of its specific course, which is different from typical myocardial ischemia [16].

This case series is remarkable since the severe symptoms occurred during every anaesthesia procedure. The female patient was resuscitated two times including therapeutic hypothermia, but fortunately no neurological or cognitive deficit was detectable.

Randomized controlled trials (RCT) are needed to establish the most effective treatment options in patients with Tako-Tsubo cardiomyopathy. Additionally, it would be very helpful to establish clinical registries on this syndrome to gain further insight into the pathogenesis and treatment in patients during anaesthesia.

## Abbreviations

ALS: Advanced Life Support
BLS: Basic Life Support
bpm: Beats per minute
BURP: Backwards upwards rightwards pressure
C&L: Cormack & Lehane
CHD: Coronary heart disease
CPR: Cardiopulmonary resuscitation
ECG: Electrocardiography
ECMO: Extracorporeal membrane oxygenation
GA: General anaesthesia
IABP: Intra-aortic balloon pump
ICU: Intensive care unit
i.v.: Intravenous
kg: Kilogram
LVOT: Left ventricular outflow tract
LVOTO: Left ventricular outflow tract obstruction
m: Meter
mg: Milligram
μg: Microgram
NIBP: Non-invasive blood pressure measurement
OR: Operating room
PEA: Pulseless electrical activity
RA: Regional anaesthesia
RCT: Randomized controlled trial
ROSC: Return of spontaneous circulation
SAM: Systolic anterior motion
TEE: Transoesophageal echocardiography probe
TTC: Tako-Tsubo cardiomyopathy
TTE: Trans-thoracic echocardiography
VF: Ventricular fibrillation
yrs: Years
